# Rabies in an imported dog, Ontario, Canada, 2022

**DOI:** 10.14745/ccdr.v49i01a01

**Published:** 2023-01-05

**Authors:** Paul Di Salvo, Maureen Anderson, Christine Fehlner-Gardiner, Francesca Di Mauro, Howard Shapiro, Anna Miranda, Heather McClinchey

**Affiliations:** 1 Toronto Public Health, Toronto, ON; 2 Ontario Ministry of Agriculture, Food and Rural Affairs, Guelph, ON; 3 Canadian Food Inspection Agency, Ottawa, ON; 4 Toronto Veterinary Emergency Hospital, Toronto, ON; 5 Ontario Ministry of Health, Toronto, ON

**Keywords:** imported dog, rabies, canine-mediated, risk assessment, animal importation, zoonoses

## Abstract

Importation of rabies-infected dogs results in significant and costly public and animal health risks. In January 2022, a dog in Ontario, Canada, which was imported from Iran in June 2021, developed rabies, leading to an extensive public health investigation and administration of rabies post-exposure prophylaxis to 37 individuals. The dog was infected with a rabies virus variant known to circulate in Iran. This is the second reported case of a rabies-infected dog imported into Canada in 2021 from a high-risk country for canine mediated rabies. This case emphasizes the need for public education regarding the risks associated with importing dogs from high-risk countries for canine-mediated rabies and the benefits of establishing a public health team specializing in rabies exposure investigations.

## Introduction

The rabies virus is primarily transmitted through saliva, most commonly via animal bites, and causes infection in mammals, including humans, that is almost invariably fatal, with the clinical course rarely lasting beyond seven days in humans ((1–3)). In humans, rabies causes an estimated 59,000 deaths annually worldwide ((4)). The majority of cases occur in rabies-endemic areas, with approximately 99% resulting from canine-mediated rabies ((4,5)). There are many variants of the virus, and these variants tend to be present in specific animal species and/or geographical locations ((6)).

Canine-mediated rabies was eliminated from the United States (US) in 2007 and has not been detected in Canada since rabies variant typing began in the 1980s ((7)). Animal importation, however, has the potential to introduce rabies and other zoonotic diseases into domestic animal and human populations. Countries such as Canada and the US have established control programs and regulations for rabies, which include rabies vaccination requirements for imported dogs, but these regulations do not always prevent the importation of infected dogs during their incubation phase: between 2015 and 2021, four dogs with canine-mediated rabies were imported into the US ((8)). In July 2021, a dog from Iran became the first reported case of canine-mediated rabies imported into Canada ((9,10)).

In July 2021, the US Centers for Disease Control and Prevention (CDC) implemented a temporary suspension of dogs entering the US from 113 countries considered high-risk for canine-mediated rabies, following a 52% increase in imported dogs from these countries being denied entry on arrival over the preceding years, mainly due to fraudulent rabies certificates ((11,12)). The temporary suspension was implemented while the CDC evaluated options to address the issue long term. It has been estimated that 23% of commercial dog imports to Canada from 2013 to 2019 originated from countries considered high-risk for canine-mediated rabies (*personal communication, Jillian Blackmore, March 11, 2022*).

In January 2022, a dog in Toronto, Canada, developed rabies following importation from Iran in June 2021. This is the second reported case of dog infected with canine-mediated rabies imported into Canada in 2021. Local public health units investigated the case and worked with human and animal health agencies to manage the risk to human health.

## Public health investigation

**Figure 1:**
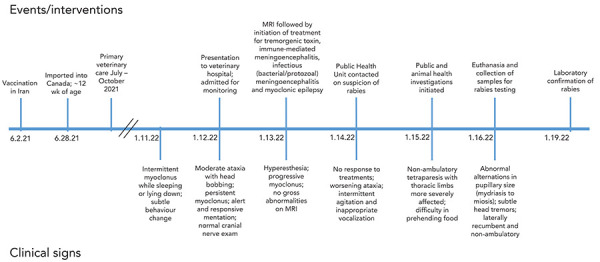
Timeline of events and clinical progression of imported rabid dog in Ontario, Canada, June 2021 to January 2022 Abbreviations: MRI, magnetic resonance imaging; wk, week

The affected dog was imported into Canada on June 28, 2021, at approximately three months of age from Tehran, Iran—a country considered high-risk for canine-mediated rabies by the Canadian Food Inspection Agency (CFIA) ((13)). It was imported by an animal rescue organization, as a personally owned pet, with documentation of rabies vaccination in Iran dated June 2, 2021, meeting the importation requirements since there is no waiting period between vaccination and importation for personally owned pets. The dog had no observed exposures to bats or other wild animals while in Canada. On January 11, 2022, the dog developed mild neurological abnormalities that progressed rapidly despite intensive in-hospital care until the dog was euthanized on January 16, 2022. Details of the dog’s clinical progression are presented in **Figure 1**.

On January 15, 2022, six Ontario public health units, led by Toronto Public Health, initiated a potential rabies exposure investigation, with the support of other agency partners, including the Ontario Ministry of Health, Ontario Ministry of Agriculture, Food and Rural Affairs, Canada Border Services Agency and CFIA.

Tissues were submitted to the CFIA laboratory for rabies testing and a positive fluorescent antibody test result was confirmed on January 19, 2022. Further testing by indirect immunofluorescence assay and reverse transcription-polymerase chain reaction confirmed the initial rabies diagnosis. Partial sequencing of the nucleoprotein gene followed by phylogenetic analysis indicated that the infecting virus grouped with canine variant rabies viruses known to circulate in Iran and Iraq.

Any person or domestic animal that had contact with the infected dog during the potential (ten days) viral shedding period, from December 31, 2021, to January 16, 2022, was interviewed to determine their rabies exposure risk. Rabies exposure risk assessments were conducted by the public health units in accordance with the Ontario Rabies Prevention and Control Protocol, 2020 in consultation with the potentially exposed persons and their healthcare providers, as necessary ((14)). A total of 37 individuals were administered rabies post-exposure prophylaxis.

A total of 42 individuals were interviewed due to having contact with the infected dog during the at-risk period, of which 41 were family or friends of the animal owners (n=16) or staff at the veterinary clinic (n=25). There was one initially unidentified Toronto resident (and their pet dog) who had contact with the infected dog during the risk period but for whom the animal owners had no means of contact. Toronto Public Health and an adjacent health unit undertook strategies to locate this individual, including animal control database searches, door-to-door canvassing over a 13-block area and a neighbourhood poster campaign. Ultimately, a media campaign was successful in reaching the individual who came forward on January 26, nearly two weeks following the exposure.

Public health staff identified seven dogs associated with individuals who were assessed for potential exposure to the rabid dog. Two of the dogs did not have any contact with the rabid dog during the risk period for virus shedding. Exposure of the other five dogs, all of which were currently vaccinated for rabies, was assessed by their primary care veterinarians and Ontario Ministry of Agriculture, Food and Rural Affairs veterinary staff. Three dogs had only minor casual contact with the infected dog and were not considered at risk for rabies exposure. Two of the remaining dogs had potential non-bite exposure to the infected dog’s saliva. One was revaccinated within seven days of exposure and was placed under observation for 45 days. Because the seventh dog was not identified until two weeks after the exposure, it was placed under a precautionary confinement period for three months following booster vaccination. None of the in-contact dogs developed rabies.

## Conclusion

This is the second reported case of a rabies-infected dog imported into Ontario, Canada in 2021. This case emphasizes the need for countries to be vigilant with animal importation regulations, and for owners of imported dogs from high-risk countries to understand that the risk of these dogs developing rabies can persist for weeks to months after arrival. While this dog arrived with documented rabies vaccination and appeared healthy, further investigation revealed significant inconsistencies that cast doubt on the validity of the documentation and likely efficacy of the reported vaccination. Federal import requirements for dogs have been under review in Canada for several years; in May 2021 various changes were made to importation requirements for commercial dogs under eight months of age ((15)). This review should continue for all categories of dogs, with the aim of preventing animals infected with rabies from entering Canada. In June 2022, the CFIA announced that as of September 28, 2022, commercial dogs from countries at high-risk for dog rabies will no longer be permitted entry into Canada, regardless of age ((13)).

This investigation highlights the need for public health agencies to ensure fulsome rabies exposure risk assessments are conducted for every reported exposure, including scrutinizing vaccination certificates and travel history of pets. Based on the animal’s estimated age at the time of rabies vaccination in Iran, the use of a vaccine product not licensed for use in Canada and provincial rabies vaccination regulations, this dog should have been revaccinated for rabies upon arrival in Ontario. It is unknown whether re-vaccination on arrival would have prevented the onset of rabies infection in this dog, particularly given the abnormally long incubation period of over six months.

Toronto Public Health led this investigation with four Public Health inspectors from a slightly larger team of Public Health inspectors with specialty training in rabies investigations. This small team was very efficient and effective at conducting patient consultations, complex risk assessments and contact tracing. This allowed other team members to focus on other routine investigations and negated the need to utilize non-specialist Public Health inspectors, despite the large number of individuals who required assessments. Where possible, public health agencies should consider establishing specialized teams of public health officials for investigating potential rabies exposures.

This investigation also highlights the ongoing need to raise awareness with healthcare providers, veterinarians, animal rescue organizations and the public, regarding the risk of rabies in imported dogs. This will help rescue organizations and animal owners to make more informed decisions on selection of animals for import and adoption, and help healthcare providers and veterinarians to better manage their respective patients. Additional actions that should be considered include ongoing work to identify high-risk rabies countries, implementing timely and legislated rabies immunization of animals and improving both qualitative and quantitative assessment of canine imports to Canada.
